# Molecular cloning and characterization of protein disulfide isomerase of Brugia malayi, a human lymphatic filarial parasite

**DOI:** 10.17179/excli2017-214

**Published:** 2017-06-01

**Authors:** Pravesh Verma, Pawan Kumar Doharey, Sunita Yadav, Ankur Omer, Poonam Singh, Jitendra Kumar Saxena

**Affiliations:** 1 Division of Biochemistry, CSIR-Central Drug Research Institute, BS10/1, Sector 10, Jankipuram Extension, Sitapur Road, Lucknow 226031, Uttar Pradesh, India; 2 Division of Toxicology, CSIR-Central Drug Research Institute, BS10/1, Sector 10, Jankipuram Extension, Sitapur Road, Lucknow 226031, Uttar Pradesh, India

**Keywords:** protein disulfide isomerase, Brugia malayi, antifilarials, homology modelling and docking

## Abstract

Lymphatic filariasis results in an altered lymphatic system and the abnormal enlargement of body parts, causing pain, serious disability and social stigma. Effective vaccines are still not available nowadays, drugs against the disease is required. Protein disulfide isomerase (PDI) is an essential catalyst of the endoplasmic reticulum which is involved in folding and chaperone activities in different biological systems. Here, we report the enzymatic characterization of a *Brugia malayi* Protein disulfide isomerase (BmPDI), which was expressed and purified from *Escherichia coli* BL21 (DE3). Western blotting analysis showed the recombinant BmPDI could be recognized by anti-BmPDI Rabbit serum. The rBmPDI exhibited an optimum activity at pH 8 and 40 °C. The enzyme was inhibited by aurin and PDI inhibitor. Recombinant BmPDI showed interaction with recombinant *Brugia malayi* calreticulin (rBmCRT). The three-dimensional model for BmPDI and BmCRT was generated by homology modelling. A total of 25 hydrogen bonds were found to be formed between two interfaces. There are 259 non-bonded contacts present in the BmPDI-BmCRT complex and 12 salt bridges were formed in the interaction.

## Introduction

Lymphatic filariasis affects about 120 million people worldwide (Michael et al., 1996[32]; Doharey et al., 2014[9]). Several excretory and secretory (E/S) products are discharged by the parasites as immunomodulatory factors, which are responsible for modulation or blockage of the effective immune response of the host (Allen and Macdonald 1998[2]; Holland et al., 2000[20]; Maizels and Yazdanbakhsh, 2003[30]; Nisbet et al., 2010[37]). Previously we have reported that calreticulin of filarial parasite differs from host and helps in establishment of infection by modulating the complement system of host (Yadav et al., 2014[61]). Diethylcarbamazine (DEC), ivermectin alone and in combination with albendazole are mainly microfilaricidal in nature and exert few effects on adult worms. Symptoms of resistance have been reported for albendazole and ivermectin (Osei-Atweneboana et al., 2007[39]; Schwab et al., 2007[47]), therefore attempts are now being continued to identify new macrofilaricides/embryostatic agents with microfilaricidal activity to reduce or eradicate the problem of emerging drug resistance. The availability of the complete genome information for *B. malayi* will help to identify of the key enzyme that can be utilized as a drug target. 

Protein disulfide isomerases (PDIs) are emerging as essential factors in health and disease in modern era (Grek and Townsend, 2014[17]). The cell surface or secretory proteins are frequently synthesized in the endoplasmic reticulum (ER) where they slip into the secretory pathway to the cell surface. Disulfide bond formation is one of the crucial steps in protein folding. PDI also facilitates proteins to acquire their correct three dimensional structure (Appenzeller-Herzog and Ellgaard, 2008[3]; Hatahet and Ruddock, 2009[18]). Misfolded proteins accumulating as large, insoluble aggregates hinder the cell function (Stefani, 2004[50]). Rapid and effective protein folding is a fundamental requirement for cell viability, and disulfide bonds maintain added stability to proteins by covalently cross-linking two cysteines that provide the appropriate protein folding and conformation which act as co-receptors for cell reorganization, and avoid cellular toxicity associated with ER stress and protein misfolding (Kimura et al., 2005[23]; Tian et al., 2004[54]). PDI has been shown to be involved in the production of the cuticle in *Caenorhabditis elegans* (Eschenlauer and Page, 2003[13]). PDI is a multifunctional protein and belongs to the thioredoxin superfamily (Hong and Soong, 2008[21]). PDI is a most abundant and very important calalyst of ER depicted as an ER-resident protein (Lambert and Freedman, 1985[25]). It is also reported that excretory/secretory (ES) proteins of schistosomes play important roles in modulating host immune systems (Hewitson et al., 2009[19]). PDI was also playing a role in *Leishmania *virulence and survival (Achour et al., 2002[1]). During cholera infection, it was reported that PDI played a role in discharge of cholera toxin (Tsai et al., 2001[55]). In case of Human Immunodeficiency Virus, PDI licence the HIV to crawl into host cells (Gallina et al., 2002[15]). A recent study showed that PDI also played a crucial roles in antigen presentation by MHC I molecules (Raghavan et al., 2008[41]). All of the nematodes are sheathed in an exoskeleton (cuticle) and undergo four cuticle moults from the L1 hatchling to the mature adult. In nematodes, non-reducible cross-links are responsible for strength and integrity of the cuticle (Page, 2013[40]; Myllyharju and Kivirikko, 2004[34]). The modification that is required to stabilise the final assembled collagen triple helix, is formed by protein disulfide isomerase (Winter et al., 2007[60]).

Ryser and co-workers were reported the potential role of PDI in the internalization of pathogens (Ryser et al., 1994[43]). Santos and co-workers hypothesized that PDI involved in the reduction of the disulfide bonds present on the parasite surface, that help in the internalization of the parasite (Santos et al., 2009[46]). Therefore, all the information received marks towards the significance of PDI in the host immune response and cell invasion process in many stages of cell function and development. In spite of this a lot has been studied about PDIs in higher eukaryotes. Limited information is available respecting PDIs in filarial worms that are important for human infections, so the present study aims to characterize this protein in *B. malayi. *Previously in our lab we have cloned, expressed and purified many important drug target proteins like *Brugia malayi* Thymidylate kinase, *Brugia malayi* Calreticulin, *Brugia malayi* Glucose 6-phosphate dehydrogenase, *Plasmodium falciparum* Purine Nucleoside Phosphorylase etc. and performed their biochemical, biophysical and inhibition studies (in detail: Doharey et al., 2016[8]; Yadav et al., 2014[61]; Verma et al., 2013[57], 2016[56]; Suthar et al., 2013[52][51]; Balaramnavar et al., 2014[6]; Singh et al., 2015[49]).

The present communication reports the cloning, expression and characterization of the recombinant PDI of *B. malayi* (rBmPDI) with the aim of understanding the differences between the host and parasitic enzyme which can be fruitfully utilized for designing of selective inhibitors with potential antifilarial activity.

## Material and Methods

### Materials

All the reagents were procured from Sigma (St. Louis, MO, USA). Ni-NTA agarose and gel elution kit were purchased from Qiagen (Germany). IPTG, pre-stained markers and restriction enzymes (*NdeI* and *XhoI*), cDNA synthesis kit and T4 ligase were purchased from MBI Fermentas (Hanover, MD, USA). Sephadex 200HR gel filtration column was purchased from GE Health- care. pET28a (+) expression vector was purchased from Novagen (Madison, WI, USA). pGEMT cloning vector and Taq DNA polymerase were purchased from Promega (Madison, WI, USA). All the animal handling was done by taking the permission of Institutional Animal Ethics Committee (IAEC) of CSIR-CDRI, Government of India.

### Maintenance of B. malayi infection

6-8 week old male *mastomys* were inoculated with 200 L3 of *B. malayi.* The infective larvae developed into adult parasites and microfilariae appeared in the blood of infected animals after 90 days. The adult parasites were collected from peritoneal cavity of infected animals in phosphate-buffered saline (PBS) (Singh et al., 2008[48]). 

### 3D structure generation, optimization and evaluation 

The 3 dimensional structure of BmPDI protein was generated through Swiss modeller based on the information gained from sequence alignment. Energy minimization of the predicted structure was done in two steps firstly, the hydrogen bonding network was optimized and then knowledge-based force fields were used to apply atomic-level energy minimization by 3Drefine (http://sysbio.rnet.missouri.edu/3Drefine). The residue profiles of the three-dimensional models were further checked using VERIFY3D (Eisenberg et al., 1997[12]). In order to assess the overall stereo chemical quality of the modelled protein, Ramachandran plot analysis was performed using the program Rampage and PROCHECK. Quality of generated models was evaluated by PROSA analysis (Laskowski et al., 1996[27]; Wiederstein and Sippl, 2007[59]). The modelled structure was then superimposed on the crystal template without altering the coordinate systems of atomic position in the template to analyse the root mean square deviation (RMSD) of the backbone atoms. The 3D x-ray structure of BmPDI has not been yet reported; hence we hope that the prediction of these structures will provide an insight to not only their individual structures but also to the BmCRT-BmPDI complex. The secondary structure predictions have been carried out by the self-optimized prediction method (SOPM) server with output width 70, the sequence of *Brugia malayi* protein disulfide isomerase was submitted in the FASTA format and the results were analysed. 

### cDNA synthesis and polymerase chain reaction (PCR)

Total RNA of *B. malayi* was isolated using the TRIzol reagent (Invitrogen) and synthesis of cDNA was carried out using cDNA synthesis kit (Invitrogen, USA). The quality of isolated RNA was checked on 1.0 % (w/v) agarose gel. About 20 ng RNA was subjected to cDNA synthesis using cDNA Cycle Kit (Invitrogen, USA). The putative BmPDI gene was PCR amplified from the cDNA of the parasite using gene-specific forward primers 5′ CATATGGATGGCATGGCAGCTATGA TTAGG 3′ having the *NdeI *restriction site and the reverse primer 5′ CTCGAGTTCAGTATGCTGTGCTTCTTCATC 3′ having the *XhoI* restriction site designed on the basis of sequence information available at http://www.kegg.jp/. PCR amplification consisted of 30 cycles (30 s at 94 °C, 1 min at 61.3 °C and 2 min at 72 °C), followed by extension cycle (10 min at 72 °C) on a PTC 200 PCR system (MJ Research, USA). The amplified BmPDI PCR product was eluted and ligated into pGEMT-easy cloning vector. *E. coli* DH5α cells were transformed with the ligated product and grown overnight on agar plates supplemented with 100 µg/ml ampicillin. Correct recombinants were identified by restriction digestion and sequencing. This recombinant plasmid (pGEMT-BmPDI) was further sub cloned in pET-28a+ expression vector system using *NdeI *and *XhoI *restriction enzymes. Positive clones were further confirmed by restriction digestion analysis. 

### Overexpression and purification of BmPDI

The *E. coli *BL-21 strain harbouring pET-BmPDI plasmid was grown overnight in Luria-Bertini (LB) medium at 37 °C, supplemented with 50 µg/ml kanamycin. Culture was induced by addition of 1 mM IPTG for overexpression of the gene of interest and grown for an additional 16-18 h at 20 °C with shaking and cells were harvested by centrifugation at 10,000 rpm for 3 min. The frozen cells were thawed in lysis buffer (50 mM NaH_2_PO_4_, 300 mM NaCl, 5 mM imidazole, 0.1 % (v/v) Tween-20 and 10 mM β-mercaptoethanol) containing protease inhibitor cocktail (Sigma) and lysed by pulse sonication (Ultrasonic processor, Model-XL-2020, Germany). The supernatant obtained after centrifugation at 10,000 ×g for 45 min was loaded on Ni^2+^-NTA column pre-equilibrated with lysis buffer. The contaminating proteins were removed by subsequent three washes with washing buffers containing (50 mM Na_2_HPO_4_, 200 mM NaCl, 30 mM Imidazole), (50 mM Na_2_HPO_4_, 200 mM NaCl, 50 mM Imidazole) and (50 mM Na_2_HPO_4_, 200 mM NaCl, 100 mM Imidazole). Recombinant protein was finally eluted by elution buffer (50 mM Na_2_HPO_4_, 200 mM NaCl, 250 mM Imidazole). For all experiments the purified BmPDI was dialyzed overnight against 50 mM Na_2_HPO_4_, 150 mM NaCl, pH 8 buffer. 

### Size exclusion chromatography (SEC) and cross-linking analysis

The native molecular mass of BmPDI was determined with the help of a calibration curve between elution volume and log molecular mass (kDa) of standard marker proteins by the Superdex 200 HR 10/300 column (manufacturer's exclusion limit 600 kDa for proteins) on AKTA FPLC (Amersham Pharmacia Biotech, Sweden). The gel filtration column was run in 50 mM sodium phosphate buffer (pH 8) containing 200 mM NaCl at a flow rate of 0.3 ml/min, with detection at 280 nm. The native molecular weight of rBmPDI was further confirmed by glutaraldehyde cross linking experiment (Bhatt et al., 2002[7]).

### Protein disulfide isomerase assay 

The BmPDI activity was measured by refolding of reduced denatured RNase A. The increase in absorbance at 260 nm, is monitored the hydrolysis of RNA catalyzed by the refolded RNase A (Lyles and Gilbert, 1991[29]). Denatured RNase A was first incubated with BmPDI (2.5 µg/mL) and sampled for 2, 5, and 7 min. into the refolding reaction. The refolded RNase A was then allowed to react with ribonucleic acid. This final reaction mixture contained 47 mM sodium phosphate, 4.7 mM EDTA, 1 mM DTT, 0.0025 % (w/v) denatured RNase A, 0.5 mM acetic acid, and 2.5 μg BmPDI in 1 mL reaction mix.

### Generation of polyclonal antibodies and Western immunoblotting

Polyclonal antibodies against rBmPDI were raised in 6 months old rabbits. 250 μg purified rBmPDI protein was emulsified with an equal volume of Freund's complete adjuvant (Sigma, USA) and injected subcutaneously in a 6-month old rabbit. Two booster doses of 150 μg rBmPDI, in Freund's incomplete adjuvant (Sigma, USA), were given after the 3^rd^ and 5^th^ week. The rabbit serum was collected after 7 days of the second booster dose and the antibody titre was measured by ELISA. Samples of 50 μg of recombinant protein were resolved on 10 % (w/v) SDS-PAGE gel and electro-blotted onto the nitrocellulose membrane (Sambrook et al., 1989[45]). The membrane was probed with anti-BmPDI serum (1:10 000 dilution) as the primary antibody, followed by HRP-conjugated antirabbit IgG as the secondary antibody. The blot was developed by chromogenic peroxidase reaction with 3,3′-diaminobenzidine (Singh et al., 2008[48]).

### Effect of pH and temperature 

To determine optimal pH, the activity was measured at different pH and effect of temperature on enzyme was measured by incubation for 10 min at different temperatures and determined the residual activity. 

### Effect of antifilarials and specific inhibitors

The effect of antifilarials viz., DEC, ivermectin, suramin, levamisole, and synthetic compounds viz., aurin and specific inhibitors of PDI bacitracin and ribostamycin on BmPDI activity were studied by incubating the enzyme with these compounds for 10 min at 25 °C and measuring the activity.

### Interaction of BmPDI with BmCRT 

#### i. Solid phase binding assay 

Microtiter plate was coated with 100 µl/well of purified recombinant BmCRT (0 to1.5µg) diluted in carbonate buffer (15 mM of Na_2_CO_3_, 35mM of NaHCO_3_, pH 9.6) for 6 h at room temperature. Control wells contained buffer and BSA. Following three washes with 0.05 % Tween 20 in PBS (PBST), the wells were blocked with 150 µl of 5 % skimmed milk in PBS for 2 h at 37 °C. 0 to 5 µg of BmPDI in 100 µl of 20 mM Tris-HCl (pH 7.4) containing 50 mM NaCl and 1 mM CaCl_2 _was added to each well and the plate was incubated at 4^ o^C overnight. The plate was washed three times with PBST and 100 µl of rabbit anti-BmPDI (at 1:1500 dilution) was added to each well. After 2 h, wells were washed once again three times as described earlier and binding was detected by probing with HRP-conjugated goat anti-rabbit IgG (at 1:3000 dilutions). The plate was kept at room temperature for 2 h followed by washing with PBST. Bound peroxides activity was measured by adding OPD (Orthophenyl diamine dihydrochloride, SIGMA) as substrate. The developed color was read at 490 nm in a microplate reader. The data are given as an average experiment ± standard deviation (SD). 

#### ii. Protein-Protein Docking

Information driven flexible docking algorithm called High Ambiguity Driven protein-protein DOCKing (HADDOCK) was used for docking the predicted three dimensional structures of CRT and PDI proteins of *B. malayi *(Dominguez et al., 2003[10]). The *ab-initio* based docking systems do not take experimental information into account, as most of the computational search and screen use experimental knowledge hence we opted the knowledge-based approach to analyse the interactions between two proteins. First of all the topology of the molecule is generated, then a rigid-body energy minimization approach is followed. Semi-flexible and flexible refinement in torsion angle space and in explicit solvent respectively is done. In each stage the structures are scored and ranked, the best structures are then carried forward to the next stage. HADDOCK uses Crystallographic and NMR system as structure calculation engine. HADDOCK performs clustering on maximum of 200 models. HADDOCK score is the weighted sum of Electrostatic energy, Van der Waals energy, Desolvation energy and Restraints violation energies. It also performs comparison to the reference structure.

### Analysing protein-protein interactions

The interactions between the predicted BmPDI and BmCRT protein model of *B. malayi* were visualised through academic version of Pymol. A separate python script “InterfaceResidues.py” was used to analyse the interacting interface residues of both the proteins. The specific protein-protein interacting residues were examined through PDBSUM (Laskowski, 2001[26]).

## Results

### Sequence analysis and 3D structure generation

The amino acid sequence alignment of *B. malayi* Protein disulfide isomerase with *C. elegans*, human, mouse, *Leishmania major*, *Leishmania Donovani, trypanosome, yeast, *and *E. coli* Protein disulfide Isomerases was carried out by Clustal W to determine the extent of their homology. Phylogenetic relationship of BmPDI with PDIs of other organisms showed that BmPDI is conserved in prokaryotes as well as in eukaryotes throughout the path of evolution. Among eukaryotic PDIs, BmPDI is closer to the *C. elegans* than human and mouse respectively and far from *leishmania, trypanosome* and *yeast.* BmPDI showed distant relationship with prokaryotic PDIs (Supplementary Figure 1). Protein disulfide isomerase of *Brugia malayi* and *C. elegans* exists as the sister node, same could be followed with *Schizosaccharomyces pombe *and *E. Coli, Mus musculus* and *Homo sapiens, Leishmania donoveni* and *Leishmania major,* whereas *Plasmodium falciparum* exists as an outgroup node (Supplementary Figure 2).

Recently a new method called the self-optimized prediction method (SOPM) has been described to improve the success rate in the prediction of the secondary structure of proteins. The sequence length was 487 amino acids whose Alpha helix (Hh) accounts 231 amino acids of about 47.43 %. The extended strand (Ee) had 82 amino acids accounting 16.84 %, Beta turn (Tt) made up of 37 amino acids making up 7.60 % and random coil (Cc) made up of 137 amino acids accounting 28.13 %. There was no 3_10_ helix (Gg), Pi helix (Ii), Beta bridge (Bb), Bend region (Ss), ambigous states and other states. The parameters were window width of 17 with a similarity threshold 8 and the number of states is 4 (Geourjon and Deleage, 1995[16]) (Supplementary Figure 3A, B, Supplementary Table 1).

Based on the information obtained from BLAST, template model was selected PDB ID: 4EKZ, the three dimensional model of BmPDI was generated. The template strand was selected based on the maximum identity score and Coverage score, and E value score; also 4EKZ is the structure of Human protein disulfide isomerase and most of the regions are conserved in both the sequences, so we opted the same as template sequence. BmCRT protein was modelled by taking 3RGO as the template sequence which showed maximum Identity score of Coverage score (Yadav et al., 2014[61]). 

**Figure 1:**
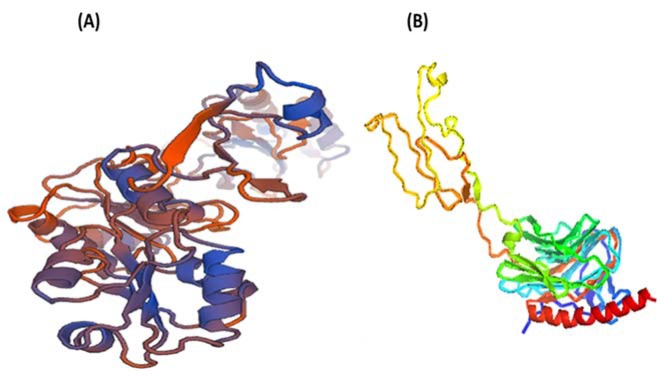
(A) 3D structure of *Brugia malayi *protein disulfide isomerase generated with the help of Swiss Modeller; (B) 3D structure of *Brugia malayi *Calreticulin generated with the help of Swiss Modeller

A three-dimensional model for BmPDI was produced by homology modelling (Figure 1(Fig. 1)). The refined model was subjected to a series of tests for its internal consistency and reliability. Comparing this result with that obtained for the human PDI, we found that the backbone conformations of our BmPDI model to be nearly as good as those of template.

The Ramachandran plot showed normal distribution of points with phi (φ) values and psi (ψ) values clustered in a few distinct regions with expected 98 % residues are in favoured region (Supplementary Figure 4). The quality of BmPDI model checked by different criteria showed that the backbone conformation (PROCHECK) and the residue interaction (PROSA II) are well within the limits established for the reliable structures and these tests suggest that a good model for BmPDI is obtained which can be further used to explore its interactions with the substrate, and inhibitors or protein-protein interaction (Supplementary Figure 5).

### Cloning, expression and purification of BmPDI

**Figure 2:**
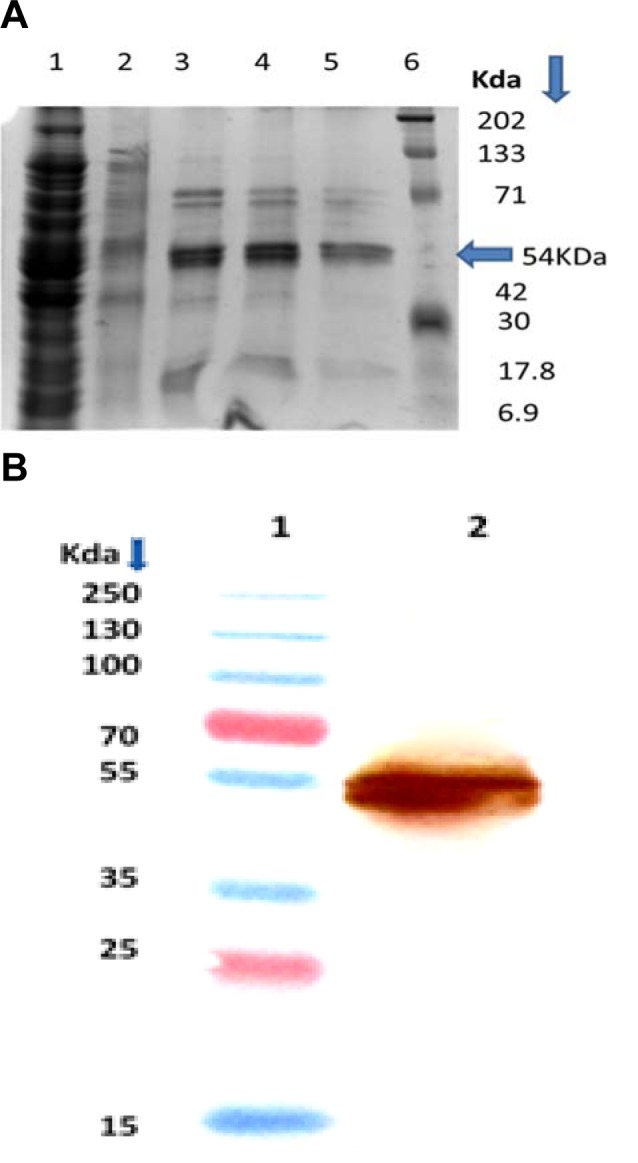
Purification of rBmPDI expressed in *E. coli.* (A) 10 % SDS-PAGE analysis of purified recombinant BmPDI. Lane 1: Flow through from Ni-NTA Column; Lane 2: Washing of the column with 50 mM imidazole; Lane 3 & 4: Elution with 200 mM imidazole; Lane 5: Elution with 250 mM imidazole. Pre-stained protein markers (Page Ruler TM, MBI, fermentas). (B) Western blot analysis using anti-BmPDI antibody as probe. Lane 1: Prestained protein marker. Lane 2: Purified protein.

*Brugia malayi* BmPDI was successfully cloned, expressed and purified by Ni^2+^-NTA affinity chromatography. The PCR product obtained by amplification of filarial cDNA was cloned into expression vector pET-28a^+ ^and subsequent transformation of this construct into *E. coli* strain Rosetta (DE3) expressed a soluble protein of expected size. The *E. coli* BL-21 strain harbouring pET-28a-BmPDI plasmid was grown overnight in Luria-Bertini (LB) medium at 37 °C, supplemented with 50 µg/ml kanamycin. Induction of expression was carried out by the addition of isopropyl d-1-thiogalactopyranoside (IPTG) to a final concentration of 1 mM and cell growth was continued at 20 °C for 16 h. Cells were harvested by centrifugation at 8000 × g for 5 min and pellet was resuspended in lysis buffer (50 mM NaH_2_PO_4_, 300 mM NaCl, 10 mM imidazole) pH 8.0 containing protease inhibitor cocktail (Sigma) and lysed by pulse sonication. Nickel affinity column (Ni^2+^-NTA) was used for single step purification of 6XHis-tagged BmPDI protein. The protein was eluted from the affinity column with 250 mM imidazole. The sub-unit molecular mass of rBmPDI was determined by 10 % SDS-PAGE according to the method of Laemmli (1970[24]). A subunit molecular mass of 54 kDa was obtained for expressed and purified BmPDI by SDS-PAGE (Figure 2A(Fig. 2)).

### Specificity of anti-BmPDI antibodies and Western immunoblotting

The titre of anti-BmPDI antibody raised against recombinant protein was found to be 1:64,000. Western immunoblotting illustrates the specificity of the antibody for the purified recombinant BmPDI (Figure 2B(Fig. 2)).

### Size exclusion chromatography and cross-linking analysis

**Figure 3:**
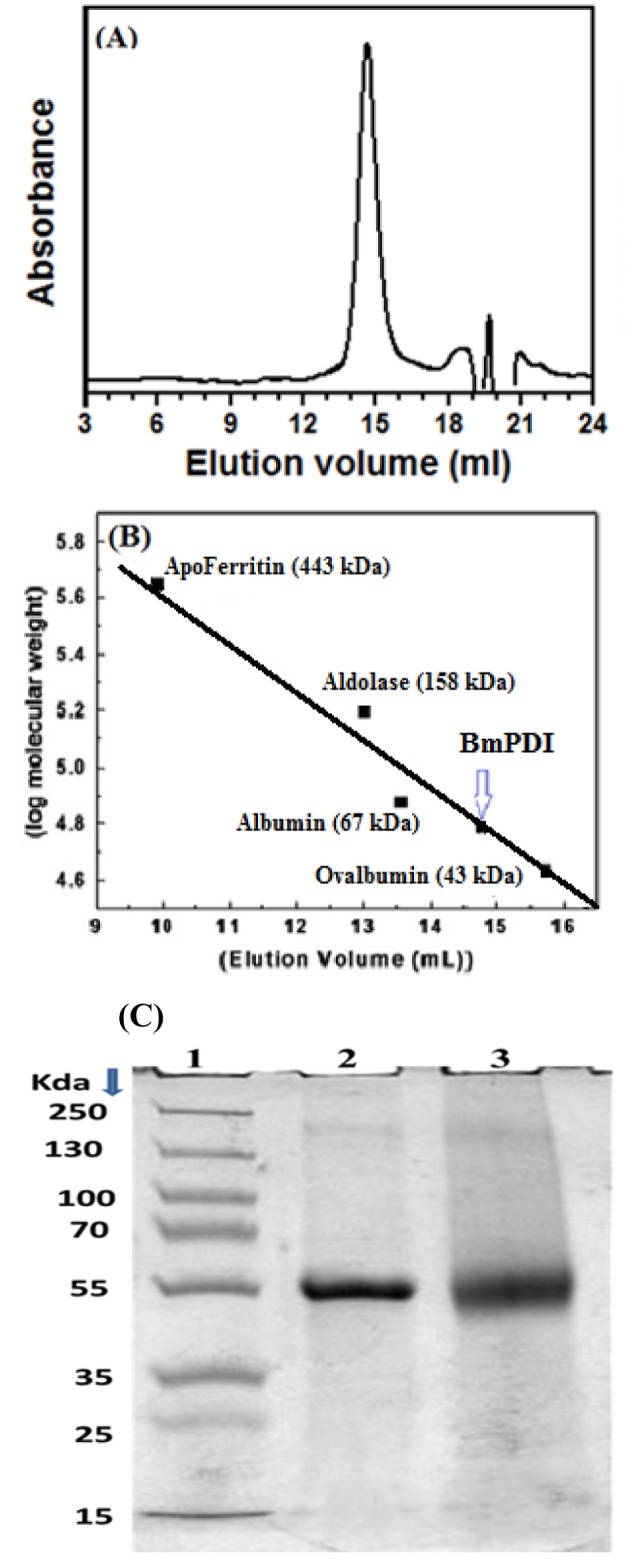
Determination of molecular mass of recombinant BmPDI. (A) Size exclusion chromatography profile of native BmPDI with absorbance at 280 nm. (B) The native molecular mass of the protein was determined by FPLC using Sephadex 200HR 10/300 gel filtration column. The column was calibrated with molecular weight standard markers: a - Ova-albumin (42.7 kDa), b - albumin (67 kDa), c - aldolase (158 kDa), and d - apoferritin (443 kDa), Arrow indicates the native molecular mass of BmPDI. (C) 10 % SDS-PAGE profile of glutaraldehyde cross-linked samples, Lane 1: Molecular weight standard markers, Lane 2: Native rBmPDI, Lane 3: Cross-linked rBmPDI with 0.1% glutaraldehyde.

The Sephadex-200 column was calibrated with standard molecular markers. The native molecular weight of rBmPDI was found to be 54 kDa as obtained by size-exclusion chromatography, indicating that rBmPDI is a monomer. Monomeric nature of BmPDI was further confirmed by interchain cross-linking of BmPDI using glutaraldehyde as cross-linker. The cross-linked product when analyzed by 10 % SDS-PAGE showed a band at 54 kDa (Figure 3A-C(Fig. 3)).

### Spectrophotometric assay of recombinant BmPDI

**Figure 4:**
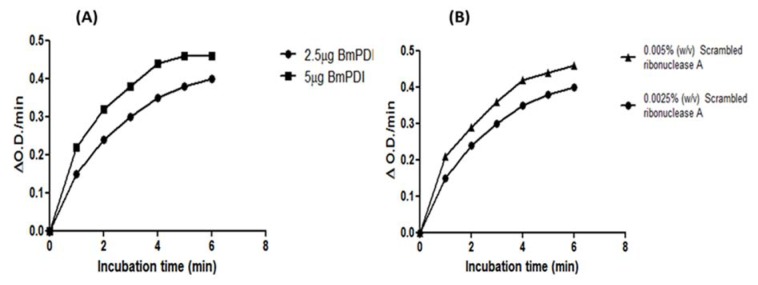
Recovery of RNase activity in the presence of rBmPDI. (A) Catalysis of folding of scrambled RNase as a measure of disulfide isomerization by rBmPDI. (B) Showing the activity of BmPDI in the presence of various concentrations of substrate. The values are the mean of three separate experiments.

As an approach to characterize the biological activity of the recombinant protein we used a spectrophotometric assay to investigate the catalyzed refolding of reduced and denatured RNase in the presence of various amounts of purified recombinant BmPDI. The results indicated that BmPDI was able to readily refold scrambled RNase, confirming its ability to catalytically rearrange the incorrect substrate disulfide bonding (Figure 4A, B(Fig. 4)).

### Optimum pH and temperature for BmPDI Activity

**Figure 5:**
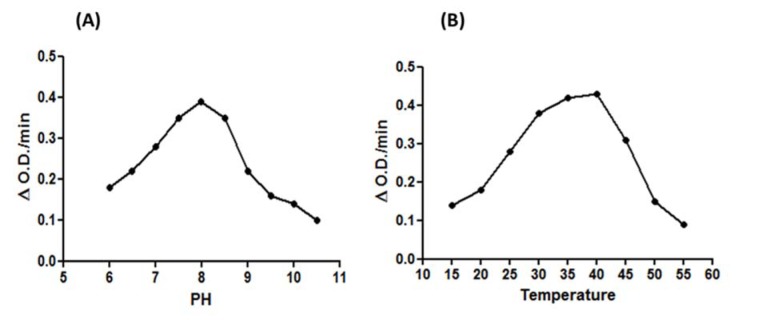
Effect of pH and temperature on the activity of BmPDI. (A) Effect of PH: the optimum activity was observed to be at PH 8. (B) Effect of temperature: the optimum temperature was found to be at 40 °C.

The effect of pH on enzyme activity was determined by using buffers of different pH (6-10.5). The recombinant enzyme exhibited activity at broad range of pH but maximum activity was observed at pH 8 (Figure 5A(Fig. 5)). The optimum temperature for enzyme activity was found to be 40 °C (Figure 5B(Fig. 5)).

### Interaction of BmPDI with BmCRT 

**Figure 6:**
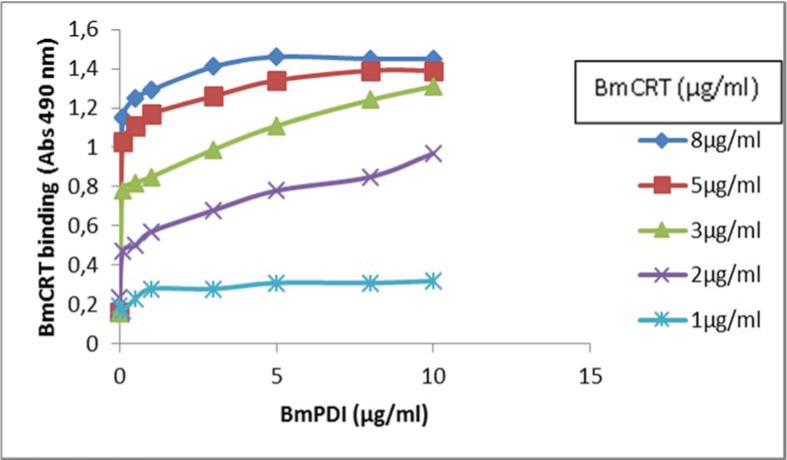
Dose dependent interaction between BmPDI and BmCRT by solide-phase binding assay. Microtiter plate was coated with 0 to 1.5 µg purified recombinant BmCRT in carbonate buffer. Control wells contained buffer and BSA. The wells were blocked with 5 % skimmed milk in PBS for 2 h at 37 °C. 0 to 5 µg of BmPDI in Tris-HCl buffer solution mentioned in method section was incubated at 4 °C overnight. The plate was washed three times and binding was detected by probing with HRP-conjugated goat anti-rabbit IgG.

The BmPDI interacts with the BmCRT in dose dependent and saturable manner. Strong binding was observed at concentration 5 µg/ml of BmPDI and 8 µg/ml of BmCRT as shown in Figure 6(Fig. 6). 

### Effect of antifilarials and specific inhibitor 

The antiparasitic compound aurin and suramin inhibited recombinant BmPDI by 80 % and 38 % at 50 µM concentration respectively, while the ivermectin, DEC, and levamisole exhibited no significant effect. The IC_50_ value for levamisole, suramin and aurin was found to be 116.29, 74.21, 17.61 µM respectively. Percentage inhibition was determined at different concentrations of inhibitors against BmPDI and IC_50_ was determined by Excel based line graphic template after plotting conc. values of each sample *vs*. % inhibition on x and y axis. 

The effect of specific inhibitor bacitracin and ribostamycin were tested on the enzymatic activity of rBmPDI. The results show that ribostamycin is more potent than bacitracin, as it inhibited the enzyme activity by 63 % and 78 % at 50 and 100 µM respectively. The bacitracin inhibited 14 and 32 % activity of rBmPDI at the same concentrations. The IC_50_ value for ribostamycin was found to be 35.64 µM (Table 1(Tab. 1)).

### Protein-protein interactions

**Figure 7:**
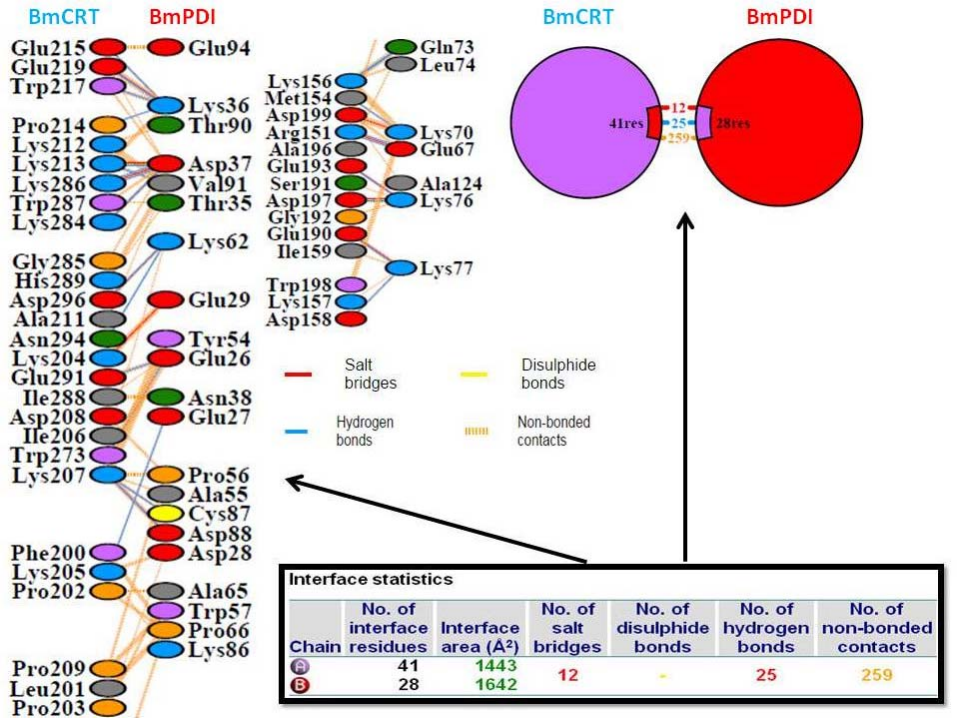
Protein-Protein Interaction. The table in the figure shows the statistics of the residues involved; the upside arrow points towards the figure pertaining gross number of residues taking part in interaction; while the arrow pointing sideways emulates the specific residues taking part in protein-protein complex formation.

Protein-Protein interactions are the most important part of the biological pathways network and important target for drug design. The binding site was obtained through literature search, where the amino acid residues 26-83 of the N-domain of PDI was found to interact with 150-240 amino acid residues P-domain of CRT protein (Yadav et al., 2014[61]) (Figure 7(Fig. 7)).

**Figure 8:**
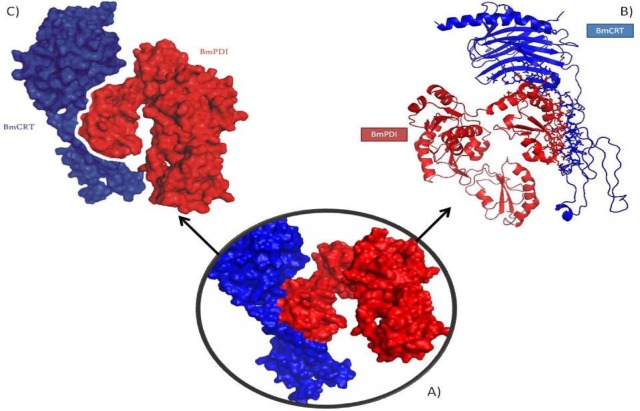
Protein-Protein interaction between modelled BmCRT (Blue) and BmPDI (Red): (A) Inset shows the associated complex with hydrophobic surface view; (B) The interacting residues represented in sticks while non-interacting part depicted in cartoon form; (C) Showing surface interactions between two proteins.

While viewing the interactions between the BmPDI and BmCRT proteins it was found that a total of 42 and 28 residues were found to be involved in interactions from BmCRT and BmPDI proteins respectively. Interface surface area of protein-protein complex was 1443 Å^2^ for BmCRT protein and 1642 Å^2 ^for BmPDI protein. A total of 25 hydrogen bonds were found to be formed between two interfaces. There are 259 non-bonded contacts present in the BmPDI-BmCRT complex and 12 salt bridges were formed in the interaction (Figure 8(Fig. 8)).

The amino acid residues mainly contributing in the interaction are shown in Figure 7(Fig. 7). In general Lys^36 ^, Asp^37^, val^91^, Lys^62^, Glu^26^, glu^27^, Cys^87^, Gln^73^, lys^70^, Lys^76^ and Lys^77^ of BmPDI were involved in hydrogen bond formation with Glu^215^, Glu^219^, Trp^217^, Pro^214^, Lys^213^, Lys ^286^, His^289^, Ala^211^, Glu^291^, Phe^200^, Lys^156^, and Lys^157^ of BmCRT. 

## Discussion

Protein folding is a complicated process which is essential to acquire biological function. Parasites have developed antioxidant defences and redox balance mechanisms for their protection from the Reactive Oxygen Species produced by host immune responses and also protect themselves against oxidative stress due to their own oxygen metabolism (Suttiprapa et al., 2008[53]; Wang et al., 2013[58]). Protein disulfide isomerases are regarded as an essential member of the extracellular anti-oxidant sword of this parasite with a crucial role in parasite-host cell interaction. A recent study reported that PDIs may also be secreted to the cell surface in some cells (Naguleswaran et al., 2005[35]). Proper folding of proteins is necessary for parasite reproduction, development and play a pivotal role in host parasite interactions. Recently it was shown that PDI is vigorously expressed in the uterus wall and oocyte of female adult *A. cantonensis* worms (Liu et al., 2012[28]). The formation and maturation of proteins occur throughout the whole life and vitellogenins are prevalent proteins in adult female nematodes that are synthesized in a large amount to support developing oocytes (Nisbet et al., 2010[37]). In nematodes, the cuticle, synthesized by the hypodermis is a necessary collagenous extracellular matrix (ECM) has fundamental role for survival of nematodes and can insulate them against adverse environmental conditions. Protein disulfide isomerase of *Brugia malayi* and *C. elegans* exists as the sister node, similar to *Schizosaccharomyces pombe, Escherichia Coli, Mus musculus, Homo sapiens, Leishmania donoveni *and* Leishmania major. *Where as in *Plasmodium falciparum* it exists as an out-group node. PDI is one of the richest proteins in the ER (Lyles and Gilbert, 1991[29]). BmPDI was expressed as a soluble protein of expected size of 54 kDa. On the other hand *Clonorchis sinensis* PDI is reported to be a monomer of 52 kDa (Hu et al., 2012[22]). Analysis of *Leishmania* genomes showed the presence of four distinct genes encoding for potential PDI proteins of 15, 40, 47 and 52 kDa that are highly conserved (Hong and Soong, 2008[21]). A 52-kDa PDI of *N. caninum* techyzoites is involved in the adhesion of parasites to host cells (Naguleswaran et al., 2005[35]). Mammalian PDI is a 57 kDa ER protein and exists as homodimer (Edman et al., 1985[11]). BmPDI purified by Ni-NTA affinity chromatography was found to be of 54 kDa. Gel filteration chromatography and glutaraldehyde cross-linking studies indicated the monomeric nature of protein. In addition to the role of PDI in proline hydroxylation, it has been also shown that PDI 2 is involved in the oxidative registration step in *C. elegans *while mutations in pdi-2 cause severe cuticle disruption and adult fecundity, whereas the mixed mutations of pdi-1, pdi-2 and pdi-3 cause embryonic lethality which clearly shows the importance of PDI in nematodes (Winter et al., 2007[60]). The results of the kinetic properties of BmPDI indicated that the enzyme is able to readily refold scrambled RNase, confirming its ability to catalytically rearrange the incorrect substrate disulfide bonding. The maximum activity was observed at pH 8 and the optimum temperature for enzyme activity was found to be 40 °C. In *Leishmania amazonensis* PDI, the optimum pH was found to be 8, while in *Entamoeba histolytica* and *Fasciola hepatica* PDI, the optimum pH was 7 and pH 8 respectively (Hong and Soong 2008[21]; Salazar-Calderon et al., 2003[44]; Ramos et al., 2011[42]). The titer of anti-BmPDI antibodies was estimated by ELISA which was found to be 1:64,000. The calreticulin interacts with protein disulfide isomerase (Baksh et al., 1995[4]). We also showed the interaction of BmPDI and BmCRT in a concentration dependent manner. The C-domain of calreticulin is acidic and binds Ca^+2^ with high capacity and low affinity, whereas the P-domain binds 1 mol of Ca^+2^ with high affinity (Baksh and Michalak, 1991[5]). While viewing the interactions between the BmPDI and BmCRT proteins it was found 42 and 28 residues were involved in interaction of BmCRT and BmPDI proteins respectively. Interface surface area of protein-protein complex was 1443 Å^2^ for BmCRT protein and 1642 Å^2^ for BmPDI protein. A total of 25 hydrogen bonds were found to be formed between two interfaces. PDI and calreticulin belong to a family of the resident ER proteins (Edman et al., 1985[11]). PDI and calreticulin have been proposed to be multifunctional proteins capable of interaction with a variety of proteins (Noiva and Lennarz, 1992[38]; Freedman et al., 1994[14]). Importantly, interaction between PDI and calreticulin leads to modulation of their functions (Baksh et al., 1995[4]). Furthermore, expression of calreticulin domains and PDI as fusion proteins with GAL4 in the yeast two-hybrid system revealed that the N and P-domain of calreticulin interact with PDI under normal cellular conditions. The interaction with PDI requires only the amino-terminal region of the N domain (amino acid residues 1- 83) of calreticulin. This is one of the most conserved regions of the amino acid sequence of calreticulins (Baksh et al., 1995[4]). As peptides and protein are two different entities and binding study with whole protein may show whole range of interactions. In full length protein only some of these sites should be surface-oriented (Naresha et al., 2009[36]). A total of 25 hydrogen bonds, 12 salt bridges and 259 non-bonded contacts were observed in the BmPDI-BmCRT complex. The effect of specific inhibitor on BmPDI showed that ribostamycin inhibited the enzyme activity by 78 % at 100 µM concentration while bacitracin did not show significant effect. Bacitracin has been shown to inhibit *Leishmania major* PDI activity, on the other hand, Ribostamycin, a bovine PDI chaperone inhibitor, did not show any inhibitory effect on *Leishmania major* PDI activity (Achour et al., 2002[1]). It was shown that bacitracin decreased* L. amazonensis* promastigote PDI activity by 50 % which further supported the view that PDIs play an important role in *Leishmania *natural pathogenicity and may serve as new target for chemotherapy (Achour et al., 2002[1]). Ramos et al. (2011[42]) reported that bacitracin can be a good inhibitor for *Entamoeba histolytica* PDI. In case of *Besnoitia besnoiti* protein disulfide isomerase (BbPDI) also showed sensitivity to bacitracin in a dose-dependent manner (Marcelino et al., 2011[31]). The antifilarials did not show significant effect on BmPDI, however suramin and levamisole inhibited the enzyme activity 38 and 30 % at 50 µM concentration respectively. The synthetic compound aurin inhibited recombinant BmPDI by 80 % at 50 µM concentration, while the ivermectin and DEC, exhibited no significant effect. BmPDI might plays a major role in correct folding of immunomodulatory and secretory proteins and isomerisation of the proteins having disulfide bonds. Disulfide bond synthesis has a crucial role in cross-linking during chitin synthesis in filarial parsites. 

## Conclusion

In the present study *B. malayi* gene encoding Protein disulfide isomerase was successfully cloned, expressed and characterized. Results of the above study indicate that it can be utilized further for study and to explore the properties of BmPDI. Aurin and PDI inhibitors showed inhibition of the rBmPDI. rBmPDI interacts with rBmCRT in dose-dependent manner, furthermore these results were also supported by docking studies. The information obtained in the present study may be exploited in the design of parasite specific protein disulfide isomerase inhibitors.

## Supplementary Material

Supplementary material

## Figures and Tables

**Table 1:**
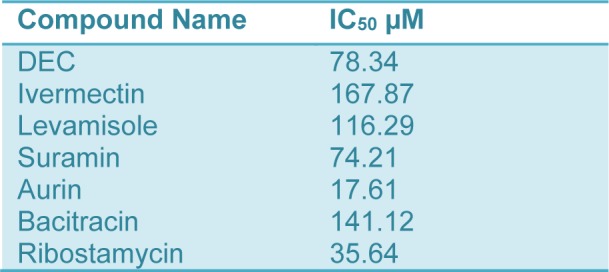
IC_50_ values of some antifilarials and specific compounds against BmPDI
