# Supplementary material for: Molecular cloning and characterization of protein disulfide isomerase of Brugia malayi, a human lymphatic filarial parasite
Source: EXCLI J. 2017 Jun 1;16:824–39. doi: 10.17179/excli2017-214 (PMC5547380; doi:10.17179/excli2017-214)
Supplement: Supplementary material [file EXCLI-16-824-s-001.pdf]

EXCLI Journal 2017;16:824-839 – ISSN 1611-2156
Received: February 13, 2017, accepted: April 11, 2017, published: June 01, 2017

Supplementary information to:

MOLECULAR CLONING AND CHARACTERIZATION OF PROTEIN DISULFIDE ISOMERASE OF *BRUGIA MALAYI*, A HUMAN LYMPHATIC FILARIAL PARASITE

Pravesh Verma[1], Pawan Kumar Doharey[1], Sunita Yadav[1], Ankur Omer[2], Poonam Singh[2], Jitendra Kumar Saxena[1]*

[1] Division of Biochemistry, CSIR-Central Drug Research Institute, BS10/1, Sector 10, Jankipuram Extension, Sitapur Road, Lucknow 226031, Uttar Pradesh, India
[2] Division of Toxicology, CSIR-Central Drug Research Institute, BS10/1, Sector 10, Jankipuram Extension, Sitapur Road, Lucknow 226031, Uttar Pradesh, India

* Corresponding author: Professor (Dr.) Jitendra Kumar Saxena, Division of Biochemistry, CSIR-Central Drug Research Institute, BS10/1, Sector 10, Jankipuram Extension Sitapur Road, Lucknow-226031,Uttar Pradesh, India.
E-mails: jksaxena@cdri.res.in, jkscdri@yahoo.com

http://dx.doi.org/10.17179/excli2017-214

This is an Open Access article distributed under the terms of the Creative Commons Attribution License (http://creativecommons.org/licenses/by/4.0/).

EXCLI Journal 2017;16:824-839 – ISSN 1611-2156
Received: February 13, 2017, accepted: April 11, 2017, published: June 01, 2017

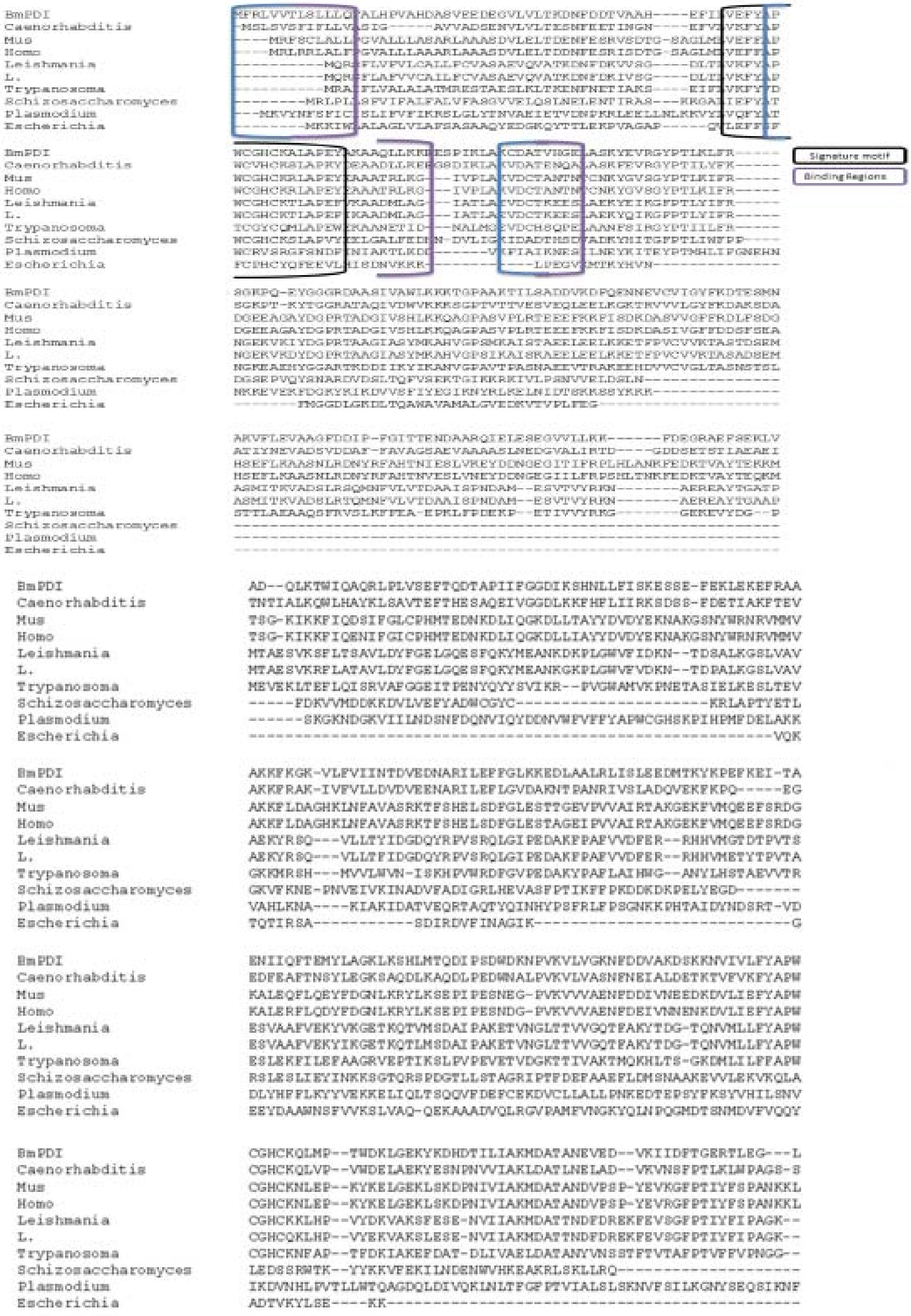

Supplementary Figure 1: Multiple alignment of the predicted amino acid sequences of Protein disulfide isomerases from *Brugia malayi* (XP_001899304.1), *Homo sapiens (*NP_005304), *Caenorhabditis elegans* (NP_497746), *Mus musculus (*NP_031978)*, Schizosaccharomyces pombe (*NP_593584)*, Leishmania major (*XP_001687289)*, Leishmania donovani* (XP_003865838), *Plasmodium falciparum (*XP_001348023)*, Trypanosoma brucei brucei (*XP_823057)*, Escherichia coli (*NP_418297) were carried out by Clustal W program (http://www.ch.embnet.org/software/ClustalW.html) and absence of amino acid is shown by dash '(-)' in the sequences while the conserved and semi-conserved residues are shown in boxes. Signature motif and binding region were also shown.

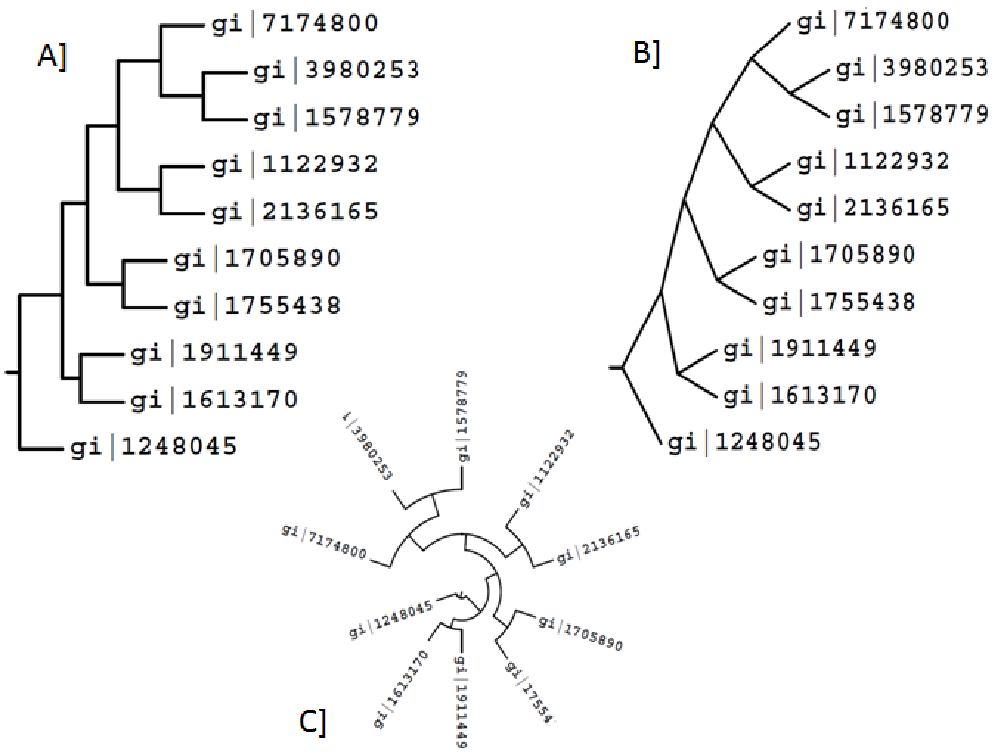

Supplementary Figure 2: Phylogenetic analysis of PDI protein sequence of *B. malayi* with PDI sequences of other organisms using PHYLIP*:* **A**) Phenogram of the sequences of PDI protein from different organisms; **A**) Phenogram; **B**) Cladogram; **C**) Circular tree [gi|170589085: BmPDI; gi|16131701: *Escherichia coli* str. K-12 substr. MG1655; gi|112293264: *Mus musculus*; gi|21361657: *Homo sapiens*; gi|157877997: *Leishmania major* strain Friedlin; gi|398025354: *Leishmania donovani*; gi|19114496: *Schizosaccharomyces pombe*; gi|17554386: *Caenorhabditis elegans*; gi|71748004: *Trypanosoma brucei brucei*; gi|124804507: *Plasmodium falciparum*].

EXCLI Journal 2017;16:824-839 – ISSN 1611-2156
Received: February 13, 2017, accepted: April 11, 2017, published: June 01, 2017

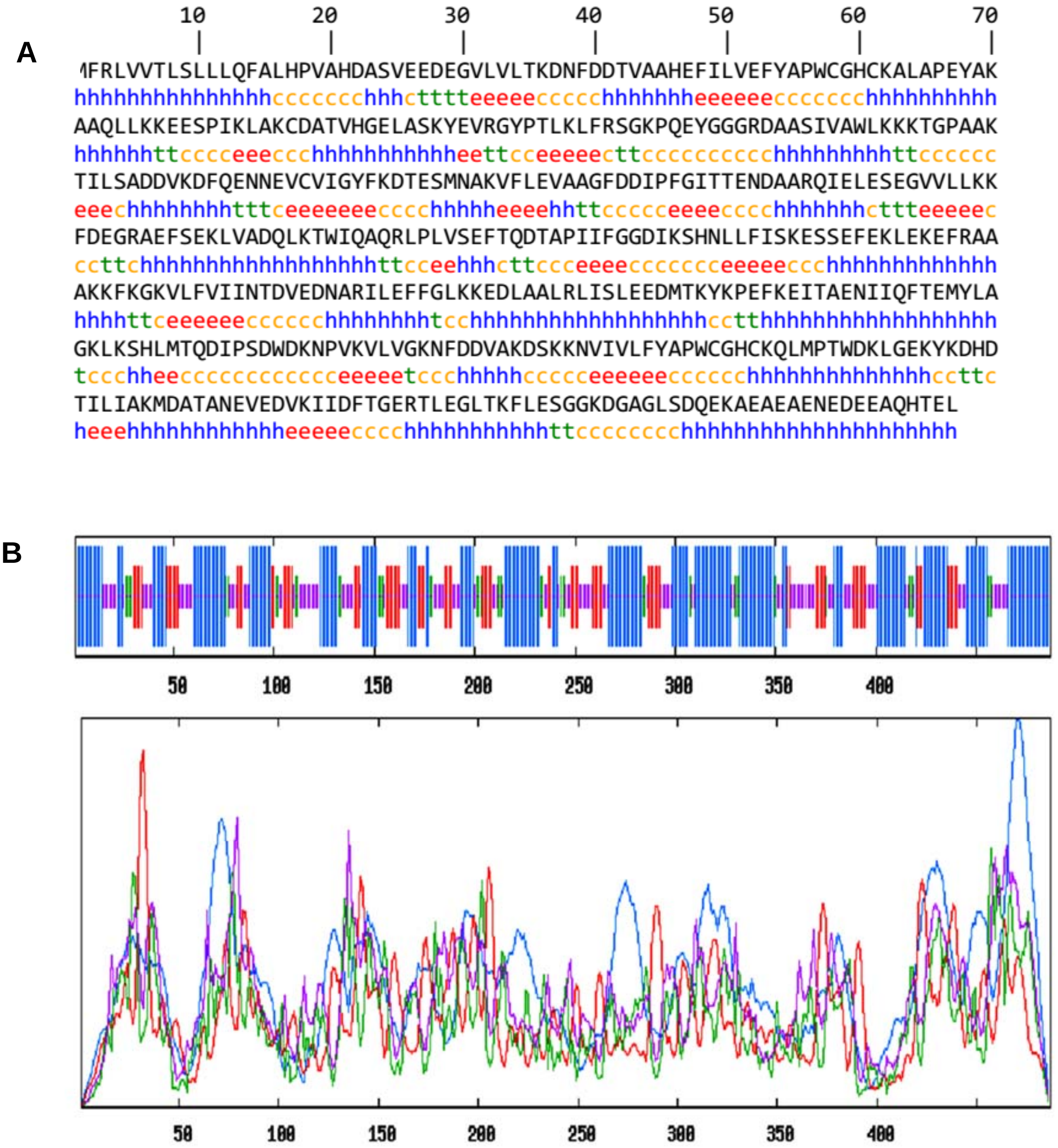

Supplementary Figure 3: (A) Secondary structure of *Brugia malayi* Protein disulfide isomerase predicted by using SOPMA C= Random coil, H= Helix, E= extended strand. **(B)** Predicted secondary structure of *Brugia malayi* Protein disulfide isomerase. Here, helix is indicated by blue, while extended strands and beta turns are indicated by red and green, respectively.

EXCLI Journal 2017;16:824-839 – ISSN 1611-2156
Received: February 13, 2017, accepted: April 11, 2017, published: June 01, 2017

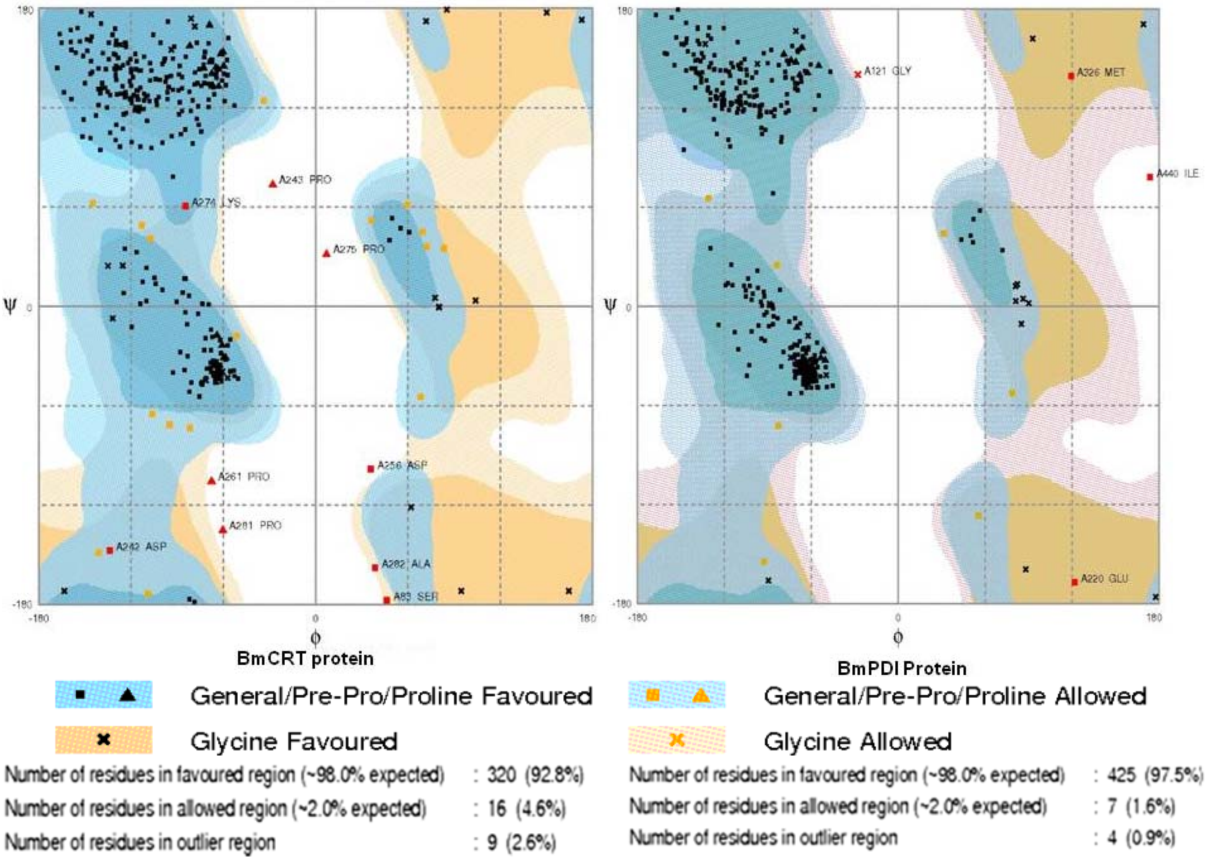

Supplementary Figure 4: Ramachandran plot of modelled protein BmPDI & BmCRT depicting most of the residues falling in the allowed region.

EXCLI Journal 2017;16:824-839 – ISSN 1611-2156
Received: February 13, 2017, accepted: April 11, 2017, published: June 01, 2017

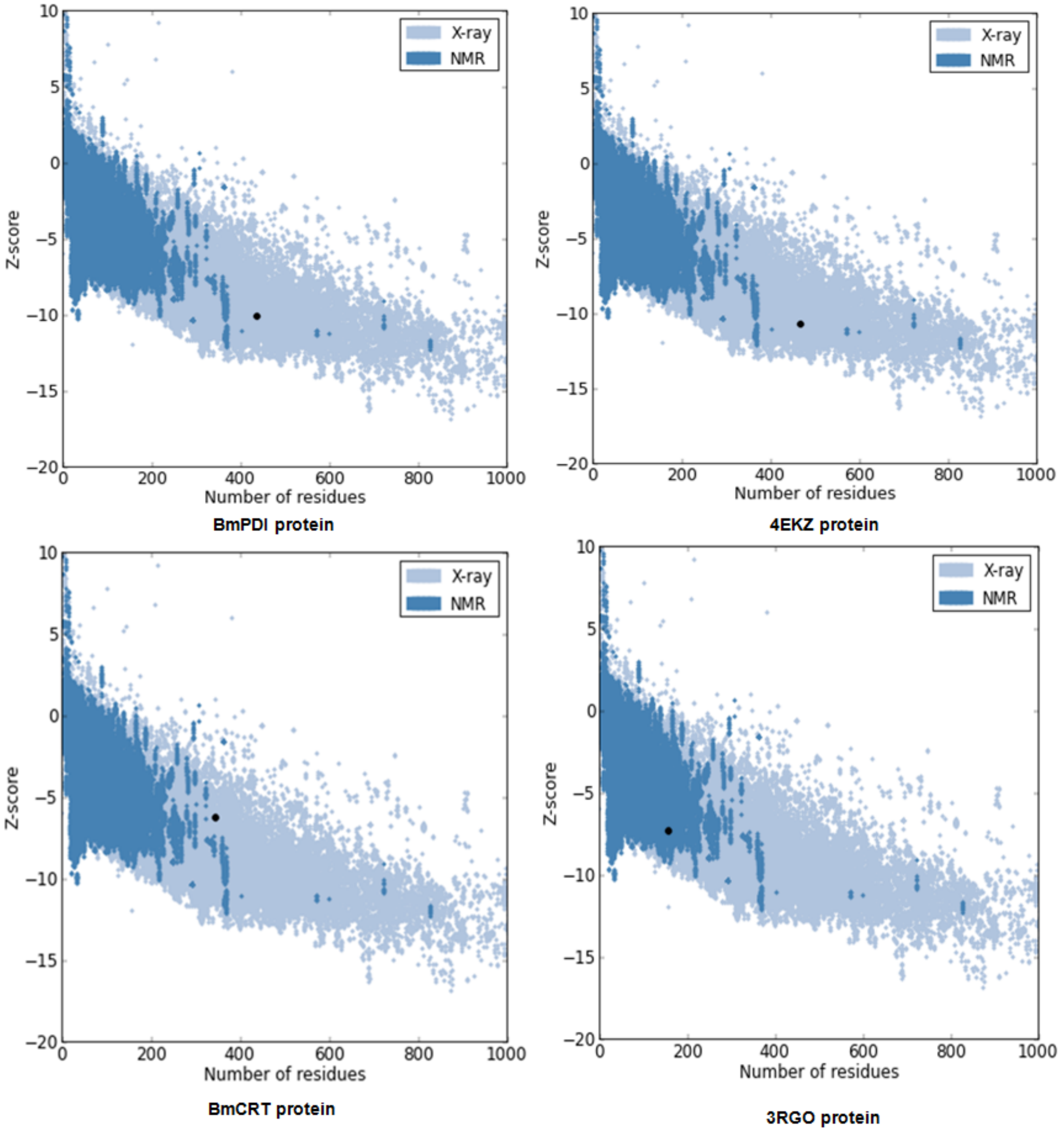

Supplementary Figure 5: Comparison of template PDB structures with modelled structures of BmCRT and BmPDI by using PROSA server.

EXCLI Journal 2017;16:824-839 – ISSN 1611-2156
Received: February 13, 2017, accepted: April 11, 2017, published: June 01, 2017

Supplementary Table 1: Representing the calculated secondary structure elements of *Brugia malayi* Protein disulfide isomerase SOPMA

**Alpha helix (Hh)**	**47.43 %**
**3_{10} helix (Gg)**	0.00 %
**Pi helix (Ii)**	0.00 %
**Beta bridge (Bb)**	0.00 %
**Extended strand (Ee)**	16.84 %
**Beta turn (Tt)**	7.60 %
**Bend region (Ss)**	0.00 %
**Random coil (Cc)**	28.13 %
